# Neurexophilin 4 is a prognostic biomarker correlated with immune infiltration in bladder cancer

**DOI:** 10.1080/21655979.2022.2085284

**Published:** 2022-06-26

**Authors:** Xianchao Sun, Shiyong Xin, Liang Jin, Ying Zhang, Lin Ye

**Affiliations:** a Department of Urology, Shanghai East Hospital, School of Medicine, Tongji University, Shanghai, China; b Department of Urology, The Second Affiliated Hospital of Anhui Medical University, Hefei, China

**Keywords:** Bladder cancer, NXPH4, prognosis, immune infiltration, progression

## Abstract

Recent studies have shown that NXPH family member 4 (NXPH4) plays an important role in the progression of cancer. However, the potential role of NXPH4 in bladder cancer (BCa) remains to be explored. The purpose of the present study was to identify whether NXPH4 could be used as a biomarker to predict the prognosis of BCa. We first examined the expression of NXPH4 in pan-cancer, and then focused on BCa. Univariate and multivariate Cox regression analysis were used to investigate whether NXPH4 could be used as an independent prognostic indicator. Gene set enrichment analysis (GSEA) was used for functional analysis of NXPH4-related genes. CIBERSORT algorithm was used to calculate immune cell infiltration levels with different NXPH4 expression. Finally, the expression of NXPH4 was validated in clinical tissue specimens and bladder cancer cell lines by immunohistochemistry and qRT-PCR. The tumor-promoting effects of NXPH4 were further investigated using counting kit-8 (CCK-8), colony formation, EdU assays, and tumor xenograft model. Our results showed that NXPH4 was highly expressed in BCa tissues. Patients in the high NXPH4 expression group had shorter overall survival (OS) and progression-free survival (PFS). We found that immune-related pathways were enriched in NXPH4-related genes. Immune cell infiltrations in BCa were also associated with different NXPH4 expression. NXPH4 was further found to be highly expressed in our validation specimens. The proliferative effect of NXPH4 was confirmed in BCa *in vivo* and *in vitro*. Overall, NXPH4 is a biomarker for predicting BCa prognosis and associated with immune infiltration.

**Abbreviations:** NXPH4: Neurexophilin 4; BCa: Bladder cancer; TCGA-BLCA: The Cancer Genome Atlas Urothelial Bladder Carcinoma; shRNA: short hairpin RNA; NC: Negative control; OS: Overall survival; PFS: Progression-free survival; TME: Tumor microenvironment; IPS: immunophenoscore; ICIs: Immune checkpoint inhibitors; DEGs: Differential expression genes.

## GRAPHICAL ABSTRACT

**Figure:**
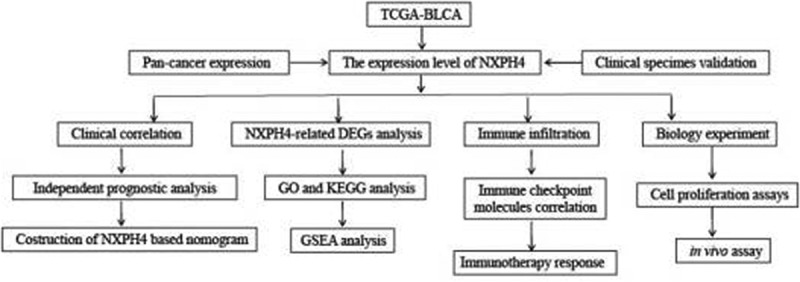

## Highlights

- NXPH4 could serve as an independent prognostic factor for predicting the prognosis of BCa patients.
- NXPH4 expression was positively related to immune cell infiltration in BCa.
- Knock-down NXPH4 could inhibit BCa growth.
- NXPH4 may be a novel target of immunotherapy in BCa.

## Introduction

Bladder cancer (BCa) is one of the common malignancies worldwide, with approximately 430,000 new cases and 165,000 deaths each year [1]. In China, the incidence and mortality rates of BCa are also on the rise. According to statistics, there were about 80,500 new cases and 32,900 deaths in 2015 [2]. Currently, the main treatment for BCa is surgery, however, the OS rate of BCa patients is not ideal due to the high recurrence and metastasis rates [3]. Therefore, it is necessary to understand the molecular mechanisms of BCa and identify effective biomarkers to develop novel therapeutic targets.

Neurexophilin 4 (NXPH4) is a neurosynaptic secretory protein that belongs to the Neurexophilin (NXPH) family. Its family protein was first thought to be a neuroglycoprotein with a mature peptide molecular weight of 29 kD [4–6]. NXPH binds to neuronal membrane proteins (NMPs) Neurexin Iɑ tightly and abundantly expressed in the nervous system. In the current state of research, there are no conclusive findings about NXPH4ʹs underlying function. Yang et al. have reported that NXPH4 was highly expressed in non-small cell lung cancer [7]. Evidences also indicated that NXPH family proteins were significantly increased in glioma, breast cancer, and pancreatic cancer [8–11].

The important role of the tumor microenvironment (TME) has been largely reported [12]. Synergistic interactions between cancer cells and their supporting cells result in malignant phenotypes of cancer, such as immortalized proliferation, resistance to apoptosis, and evasion of immune surveillance [13]. Thus, the TME can significantly influence the clinical outcome of cancer patients. The components of TME are mainly stromal cells and immune cells. Immune cells infiltrating in the peritumoral stroma exhibit an obviously characteristic of many solid tumors [14]. It has been reported that CD3^+^ and CD68^+^ cells infiltration may affect the prognosis of patients with muscle-invasive BCa [15]. In addition, the proportion of tumor-infiltrating immune cells were closely associated with the efficacy of neoadjuvant chemotherapy for BCa [16]. Transcriptome sequencing and genomic functional analysis have elucidated the role of different cell types in the dynamic regulation of TME components [17].

In this study, we aim to evaluate the prognostic value of NXPH4 in BCa and its relationship with the immune components of the TME. Specifically, we compared the expression levels of NXPH4 mRNA in tumors and corresponding normal tissues. Then, the differential expression of NXPH4 in BCa cells versus normal bladder epithelial cells was verified. The relationship between NXPH4 expression levels and survival time and clinicopathological variables of BCa patients were demonstrated. Subsequently, GSEA was performed to identify signaling pathways associated with NXPH4 regulatory mechanisms [18]. CIBERSORT algorithm was used to determine the proportion of immune cell types infiltrating in BCa patients and clarify the association between NXPH4 and immune microenvironment [19]. These studies suggest that NXPH4 is closely related to tumor-infiltrating immune cells and can be used as a biomarker for BCa diagnosis and prognosis. Moreover, we validated the biological function of NXPH4 *in vitro and in vivo*. In agreement with bioinformatic analysis, we confirmed that NXPH4 acts a tumor-promotion role in BCa and the proliferation of BCa cells were significantly inhibited after knockdown of NXPH4. In summary, this study was designed to investigate the prognostic role of NXPH4 in BCa for OS and its relationship to immunity. Based on our findings, we anticipated providing a candidate therapeutic method for BCa treatment in the future.

## Materials and methods

### Data collection

NXPH4 expression data of Genotype-Tissue Expression (GTEx) and pan-cancer were acquired from TIMER2.0 database (http://timer.comp-genomics.org/) [20]. The RNA-seq data and clinical survival information of BCa samples were obtained from The Cancer Genome Atlas Urothelial Bladder Carcinoma (TCGA-BLCA) (https://portal.gdc.cancer.gov/) [21]. The information is shown in supplementary Table S1.

### Identification of NXPH4-related genes

BCa samples were divided into low and high expression groups according to the median expression value of NXPH4, and the differential expression genes (DEGs) between the two groups were analyzed using ‘limma’ *R* package, with *p*-value < 0.05 and |log2(Fold Change)| > 1 cutoff [22].

### Functional analyses

Gene ontology (GO) and Kyoto Encyclopedia of Genes and Genomes (KEGG) enrichment analyses were conducted using *R* package ‘clusterProfiler’ to explore the biological functions and signaling pathways of NXPH4 -related genes [23].

### Immune infiltration cells analysis

In order to investigate the immune landscape with NXPH4 expression, we calculated the degree of immune cell infiltration among the cohort of TCGA-BLCA patients by CIBERSORT calculation [24]. The Wilcoxon signed-rank test was then used to analyze the difference in tumor-infiltrating immune cells between different groups and the results were visualized as a box chart. The ESTIMATE algorithm was used to calculate immune and stromal scores between two groups [25].

### The ability of NXPH4 to evaluate response to clinical treatment

Due to the critical role of immune checkpoint molecules in immunotherapy, we analyzed the correlation between NXPH4 and immune checkpoint molecules and visualized data as a correlation heatmap. In addition, immune checkpoint inhibitors have used in clinical treatment to enhance the anti-tumor immunity. We therefore predicted the clinical responses of immunotherapy between low and high expression NXPH4 groups. An immunophenoscore (IPS) was used to represent tumor immunogenicity on a scale from 0 to 10. Higher IPS scores represent increased immunogenicity. The IPS of TCGA-BLCA patients was obtained from the Cancer Immunome Atlas (TCIA) (https://tcia.at/home) [26]. Moreover, the IC50 (half maximal inhibitory concentration) of some important anti-tumor drugs were also calculated in the two groups. Differences in the IC50 were identified with ‘pRRophetic’ and ‘ggplot2’ tools in the *R* environment [27].

### Patients and tissue samples

A total of 67 paired BCa tissues and adjacent non-tumor tissues were obtained from patients who received surgery at the Second Affiliated Hospital of Anhui Medical University. None of the patients received preoperative therapy. All samples were confirmed by experienced pathologists. Tissues were snap-frozen in liquid nitrogen after resection and stored at −80°C. Written informed consents were provided by participants. This study was approved by the Ethics Committee of the Second Affiliated Hospital of Anhui Medical University. The patient information is shown in supplementary Table S2.

### RNA extraction and quantitative real-time polymerase chain reaction (qRT-PCR)

For the qRT-PCR assay, TRIzol (Invitrogen, USA) was used to extract the total RNA. GAPDH was used as an internal control. Fold-changes were calculated by the 2^−∆∆Ct^ method. Primer information is shown in supplementary Table S3.

### Cell proliferation assay

A total of 1 × 10^3^ cells were grown in each well of a 96-well plate. Cell viability was calculated with the CCK-8 system. The optical density (OD) value per well was measured at 450 nm (BioTek, USA).

### Colony formation assay

A total of 500 cells were grown in each well of 6-well plate for approximately 2 weeks until colony formation was evident. Then, the cells were fixed, stained, and photographed.

### EdU proliferation assay

To measure cell proliferation, cells were cultured in 24-well plate and treated with 5-ethynyl-2’-deoxyuridine (EdU) for 4 h according to the protocol of EdU Kit (RiboBio, China).

### Cell transfection

The immortalized human normal bladder epithelial cell line (SV-HUC-1) and bladder cancer T24, 5637 and UMUC3 cell lines were obtained from the Chinese Academy of Sciences (Shanghai, China). For cell transfection, T24 and UMUC3 cell lines at 60–70% confluence were planted in six-well plates. The short hairpin RNAs (shRNAs) against NXPH4 (sh-NXPH4) and negative control (NC) were designed and synthesized by GenePharma (Shanghai, China).

### In vivo *experiment*

Male BALB/C nude mice were purchased from Weitong Lihua Experimental Animal Center (Beijing, China) and kept in an SPF-grade pathogen free research center. This animal experiment was approved by the Animal Research Ethics Committee of the Second Affiliated Hospital of Anhui Medical University. A total of 1 × 10^6^ negative control and transfected T24 cell lines were injected subcutaneously of nude mice. The volume of the tumor was calculated as follows: Volume (mm^3^) = 0.5 × width^2^ × length. Finally, tumor tissues were carefully resected, photographed and detected using hematoxylin and eosin (H&E) and immunohistochemistry.

### Immunohistochemistry (IHC)

Tissue samples were fixed and cut into 4-μm slices. During dewaxing, rehydration, and antigen retrieval, sections were incubated with primary antibodies against Ki-67 (Abcam, USA). Images were obtained with a microscope.

### Statistical analysis

Bioinformatic analyses were conducted using *R* version 4.1.1. Student’s t test or the Wilcoxon test were used to compare continuous data. Survival rates were assessed using Kaplan-Meier (K-M) curves and the log-rank test. All statistical *p*-values were two-sided, and *p* < 0.05 was considered statistically significant.

## Results

We revealed the role of NXPH4 in BCa for the first time. NXPH4 expression was confirmed to be closely related to prognosis and immune infiltration in BCa after a comprehensive analysis. We further validated the biological function of NXPH4 *in vitro* and *in vivo*. Consequently, NXPH4 could provide a prognostic indicator of OS in BCa as it is associated with immunity and promotes tumor growth.

### NXPH4 is overexpressed in BCa

**Figure 1.**
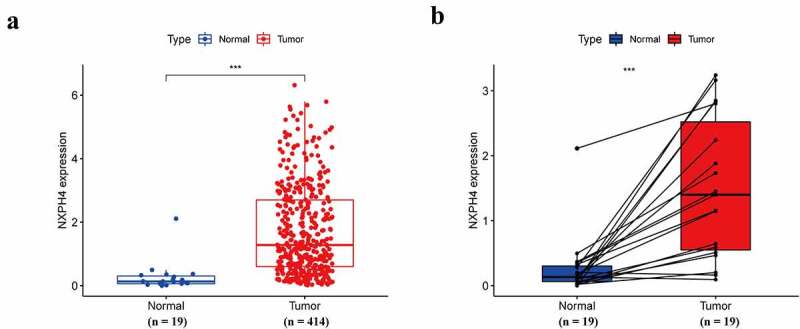
NXPH4 is overexpressed in BCa. Unpaired (a) and paired (b) expression data analyses indicated that NXPH4 expression was markedly higher in BCa. ***p < 0.001.

According to the RNA-seq data of the GTEx and TCGA databases, the expression of NXPH4 mRNA in 19 types of tumor tissues (including BCa tissues) were significantly higher than those in normal tissues (supplementary Fig. S1). It showed that NXPH4 expression was abnormally expressed across different cancer types. Next, unpaired and paired expression data analyses also indicated that NXPH4 expression was markedly higher in BCa tissues than in normal tissues (Figure 1(a,b)).

### NXPH4 expression affects the prognosis of BCa patients

**Figure 2.**
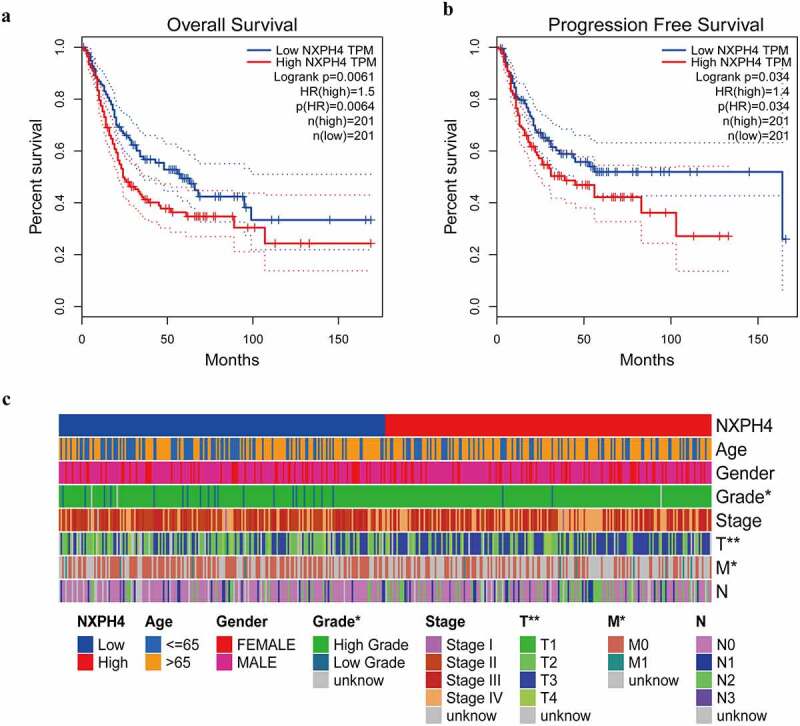
NXPH4 expression affects the prognosis of BCa patients. Patients in the high NXPH4 expression group had shorter OS (a) and PFS (b) compared with those in the low NXPH4 expression group. Correlation analysis between NXPH4 and different pathological features in BCa (c). *p < 0.05, **p < 0.01

K-M curve confirmed that NXPH4 expression was positively correlated with poor prognosis. Survival analysis of NXPH4 in BCa was conducted and the results showed that NXPH4 was closely related to OS and PFS of BCa patients (Figure 2(a,b)). Subsequently, correlation analysis performed the relationship between NXPH4 and different pathological features in BCa, and there was a significant correlation shown in Figure 2(c).

### NXPH4 is an independent risk factor for BCa

**Figure 3.**
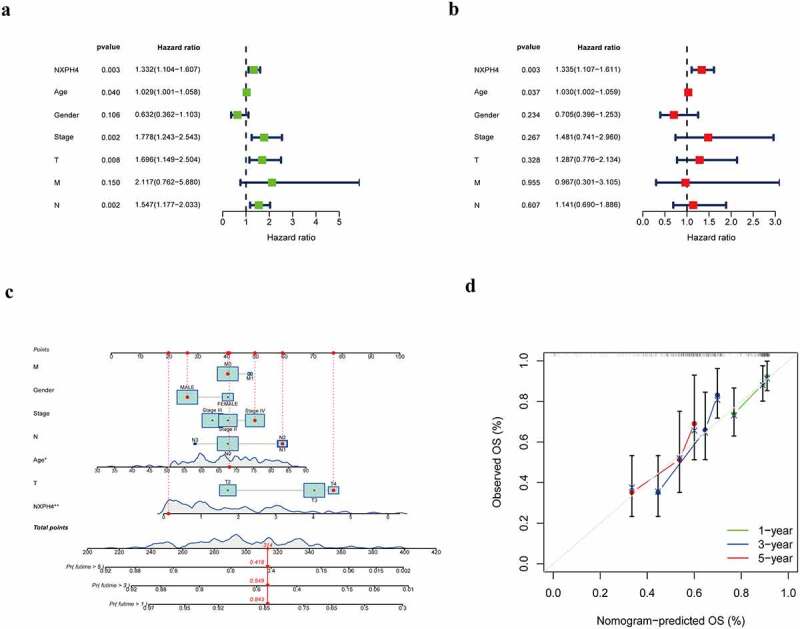
NXPH4 is an independent risk factor for BCa. Univariate (a) and multivariate (b) Cox regression analysis showed that NXPH4 was an independent risk factor correlated with the survival in BCa. A prognostic nomogram with NXPH4 and clinical variables was constructed (c). The 1-, 3- and 5-year calibration plots demonstrated the performance of the nomogram (d).

Univariate and multivariate Cox regression analysis were performed to determine independent prognosis factors. The results showed that some clinicopathological features and NXPH4 correlated with the survival of patients. Univariate Cox regression analysis showed that age, clinical stage, T stage, N stage and NXPH4 were closely related to survival. Based on multivariate analysis, only age (HR = 1.030, 95% CI = [1.002–1.059], *p* = 0.037) and NXPH4 (HR = 1.335, 95% CI = [1.107–1.611], *p* = 0.003) were significantly associated with survival. Therefore, we demonstrated that NXPH4 was an independent risk factor in BCa (Figure 3(a,b)). To evaluate the potential clinical practicality of the NXPH4, we combined the clinicopathological features and NXPH4 to construct a nomogram. As shown in Figure 3(c), a prognostic nomogram with NXPH4 and clinical variables was constructed. The 1-, 3- and 5-year calibration plots demonstrated the performance of the nomogram (Figure 3(d)).

### Biological function of NXPH4 in BCa

**Figure 4.**
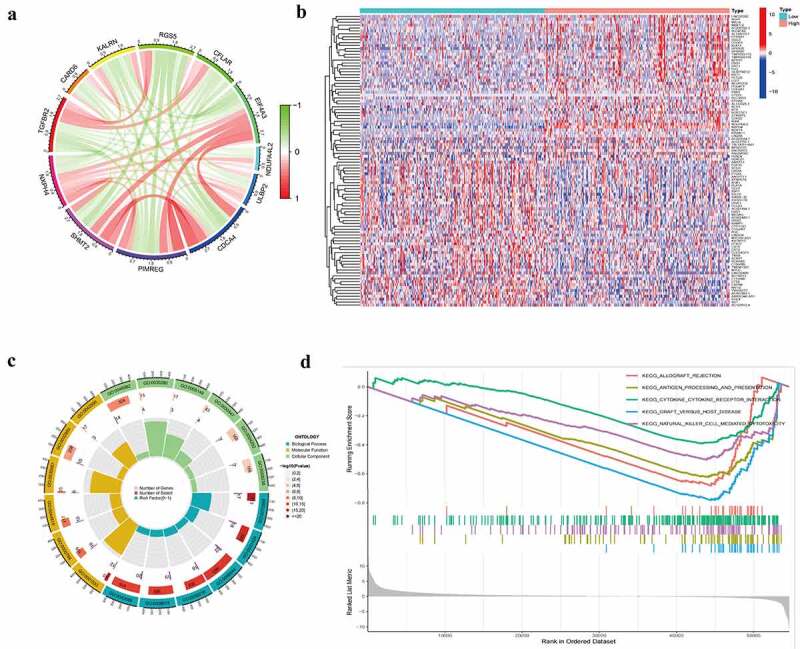
Biological function of NXPH4 in BCa. Co-expressed genes significantly associated with NXPH4 were shown as correlation circle plots (a). Red, positive correlation; green, negative correlation. The top 50 upregulated DEGs and top 50 downregulated DEGs were displayed in the heatmap (b). Red, upregulated genes; blue, downregulated genes. Enrichment analysis is shown as a circle graph (c). GSEA was used to identify signaling pathways that were NXPH4-related differentially expression genes activated in BCa (d).

Genes significantly associated with NXPH4 were identified by correlation analysis in the TCGA-BLCA dataset (supplementary Table S4), and ten co-expressed genes significant positively or negatively associated with NXPH4 were shown as correlation circle plots in Figure 4(a). NXPH4-related DEGs were used to explore the potential biological role by enrichment analysis. Based on the median expression level of NXPH4, BCa samples were divided into NXPH4-low and NXPH4-high groups. Next, we compared DEGs analysis between the two groups with |log2(Fold Change)| > 1 and adjusted *p* < 0.05. As shown in supplementary Table S5, 269 genes were screened out and identified as DEGs, including 217 upregulated genes and 52 downregulated genes. The top 50 upregulated genes and 50 downregulated genes were displayed in a heat map (Figure 4(b)). GO and KEGG analysis showed that most of these genes were linked to the events such as cell differentiation and protein heterodimerization activity (Figure 4(c), supplementary Fig. S2). Moreover, we performed GSEA using the TCGA-BLCA dataset to identify signaling pathways that were differentially NXPH4-related genes activated in BCa. The results showed that immune-related gene sets were significantly enriched in NXPH4-related DEGs (Figure 4(d)).

### Association of NXPH4 with immune cell infiltration in the TME of BCa

**Figure 5.**
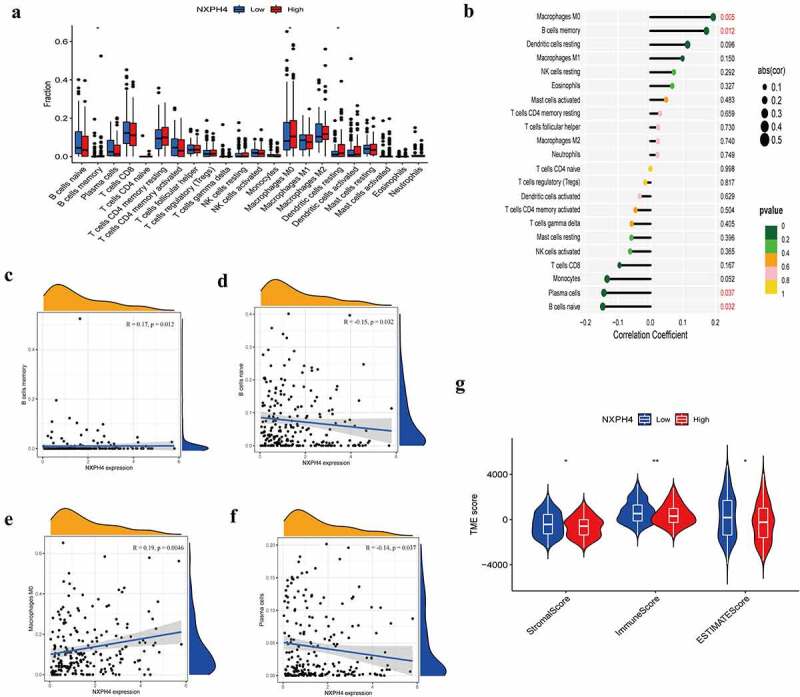
Association of NXPH4 with immune cell infiltration in the TME of BCa. The proportion of 22 immune cells infiltrating in low and high NXPH4 expression groups (a). (b) Relationships between the expression of NXPH4 and 22 types of immune infiltration cells. (c-f) The relationships between the expression of NXPH4 and memory B cells, naive B cells, M0 macrophages, and plasma cells. (g) ESTIMATE algorithm was used to investigate the correlation between the two groups in Immune scores and Stromal scores. *p < 0.05, **p < 0.01,***p < 0.001.

To further explore the relationship between NXPH4 expression and tumor immunity, we used CIBERSORT calculation to determine the proportion of 22 immune cells infiltrating in the tissues of each BCa patient. We analyzed the differences in immune infiltrating cells, and the correlation between NXPH4 expression levels and immune cell infiltration. The difference analysis revealed that the distribution of immune infiltrating cells between the two groups. Memory B cells, M0 macrophages, and resting Dendritic cells were increased in NXPH4-high expression group (Figure 5(a)). NXPH4 expression was significantly positively correlated with memory B cells and M0 macrophages, while negatively correlated with naive B cells and plasma cells (Figure 5(b–f)). In addition, other calculation methods like XCELL, TIMER, CIBERSORT-ABS, QUANTISEQ, MCPCOUNTER, and EPIC [28] were also used to compare the correlations among immune cells in different groups, suggesting there is also a difference immune status in low- and high-NXPH4 expression groups (supplementary Fig. S3). Next, the ESTIMATE algorithm was used to investigate the correlation between the two groups in Immune scores and Stromal scores. We found that the low NXPH4 expression group showed higher Immune scores and Stromal scores than high NXPH4 expression group (Figure 5(g)). These results further demonstrate that the expression level of NXPH4 can affect the immune activity of the TME in BCa.

### Role of NXPH4 in immunotherapeutic responses

**Figure 6.**
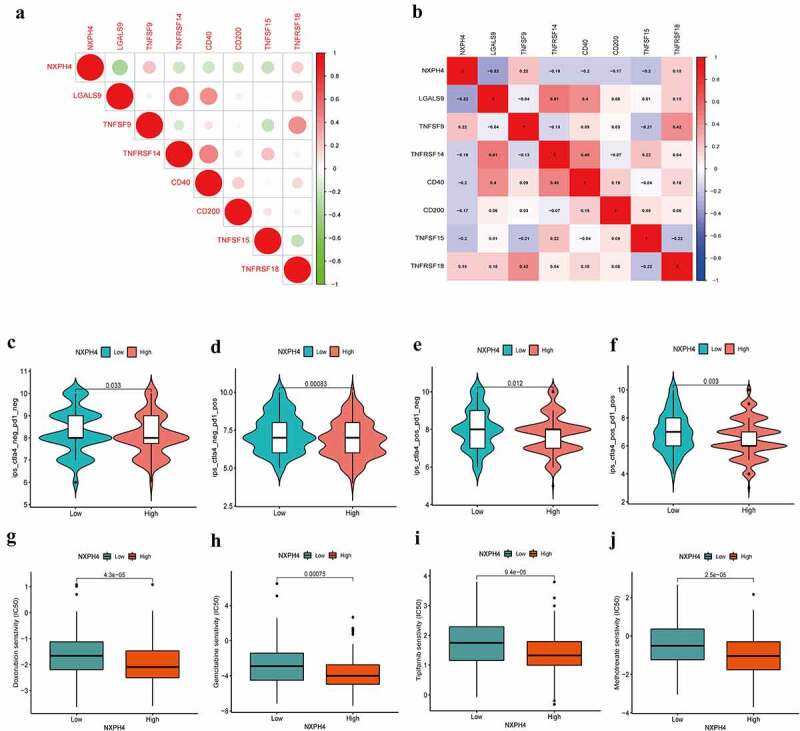
Role of NXPH4 in immunotherapeutic responses. (a-b) The association between ICIs and NXPH4. (c-f) The correlation between immunophenoscore and different NXPH4 expression groups. (g-j) Low NXPH4 expression group was positively associated with a higher IC50 of Doxorubicin, Gemcitabine, Tipifarnib, and Methotrexate.

We next analyzed the association between immune checkpoint inhibitors (ICIs) [29] and NXPH4. We found that NXPH4 was negatively associated with some ICIs such as CD40, CD200, LGALS9, and TNFRSF14 (Figure 6(a,b)). For investigating the capacity of NXPH4 predicting response to immunotherapeutic, immunophenogram analysis was undertaken to investigate association between IPS and different NXPH4 expression groups. Findings showed that low NXPH4 expression group exhibited higher IPS compared with high NXPH4 expression group, which implied that low NXPH4 expression patients exhibited higher positive response to immunotherapy (Figure 6(c–f)). Chemotherapy is an effective therapy for cancer although drug resistance can weaken the efficacy. We further analyzed the correlation between NXPH4 and chemotherapeutic efficacy. We found that low NXPH4 expression group was positively associated with a higher IC50 of Doxorubicin, Gemcitabine, Tipifarnib, and Methotrexate, indicating a different distribution of targeted IC50 agents in low and high NXPH4 expression groups (Figure 6(g–j)). Furthermore, the IC50 of some immunotherapeutic drugs also exhibited a significant differences between different groups (supplementary Fig. S4).

### *Clinical and* in vitro *validation of NXPH4 expression*

**Figure 7.**
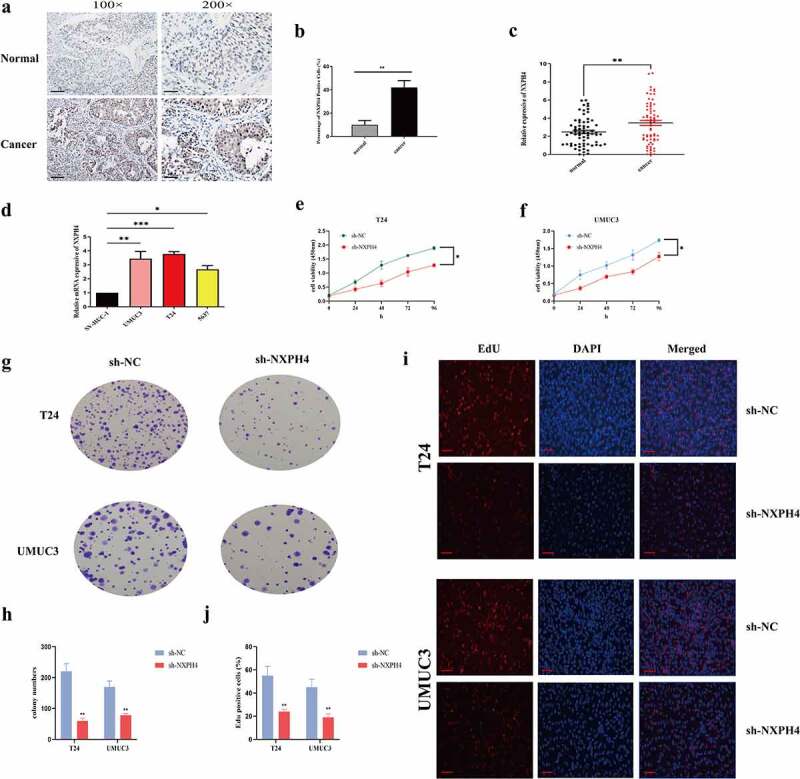
Clinical and *in vitro* validation of NXPH4 expression. (a-b) Immunohistochemistry results showed that NXPH4 were significantly highly expressed in cancer tissues. Scale bar: 100 and 200 μm. (c) The mRNA expression levels of NXPH4 were higher in cancer tissues. (d) NXPH4 was significantly increased in BCa cell lines (UMUC3, T24, and 5637) compared with SV-HUC-1. (e-f) CCK8, (g-h) colony formation, and (i-j) EdU assays showed that NXPH4 silencing inhibited the proliferation and colony formation of T24 and UMUC3 cells. Scale bar: 50 μm. *p < 0.05, **p < 0.01,***p < 0.001.

To further confirm the above results, 67 cases of BCa tissue specimens were included. Immunohistochemistry results showed that NXPH4 were significantly highly expressed in cancer tissues (Figure 7(a,b)). The results were basically consistent with the in silico analysis. Our findings also showed that the mRNA expression levels of NXPH4 were higher in cancer tissues (Figure 7(c)). Moreover, the current study showed that the levels of NXPH4 were significantly increased in BCa cell lines (UMUC3, T24, and 5637) compared with SV-HUC-1 (Figure 7(d)). Next, we analyzed the biological function of NXPH4 in BCa. CCK-8, colony formation, and EdU assays both showed that NXPH4 silencing inhibited the proliferation and colony formation of BCa cells *in vitro* (Figure 7(e–j)). These results indicated that NXPH4 may act as an oncogene in BCa.

### *NXPH4 knockdown inhibited tumorigenesis* in vivo

**Figure 8.**
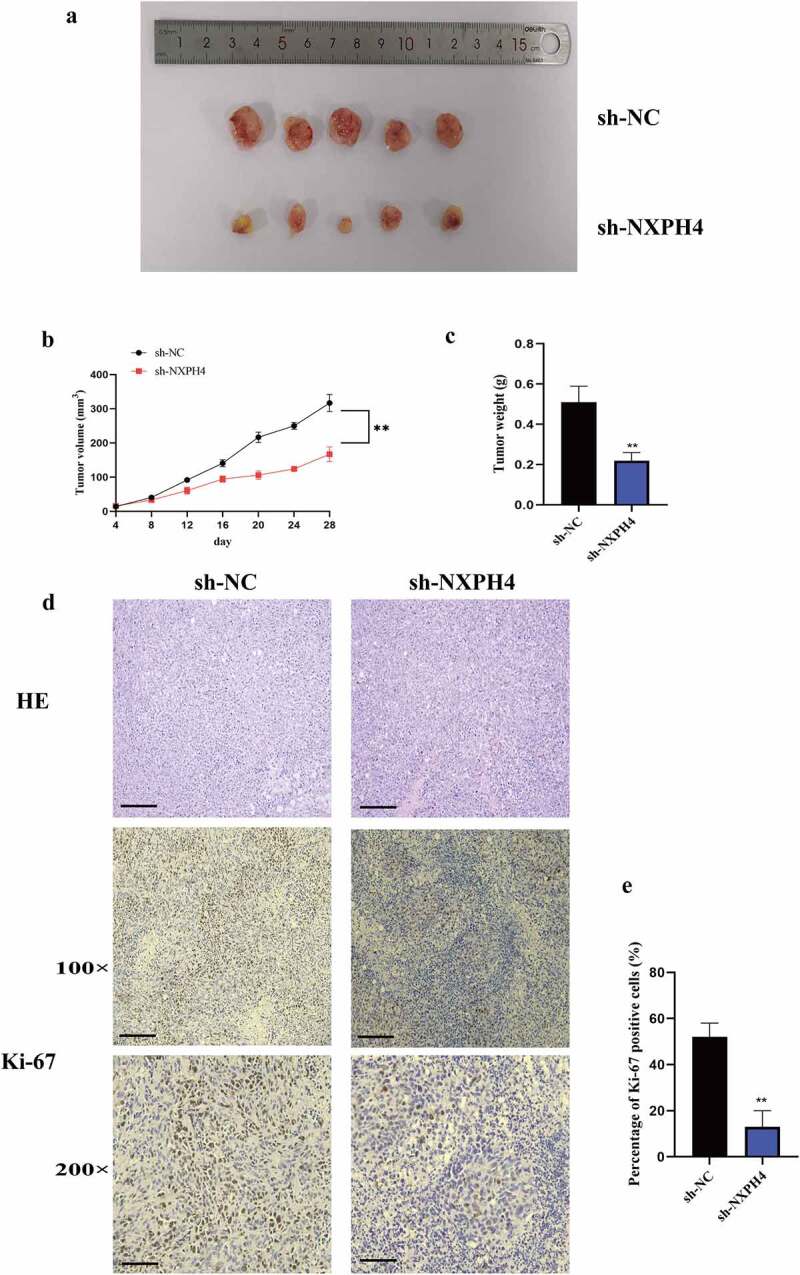
NXPH4 knockdown inhibited BCa cells tumorigenesis *in vivo*. (a) T24 cells transfected with sh-NXPH4 or sh-NC were injected subcutaneously into nude mice. (b-c) The tumor volume and tumor weight were lower in sh-NXPH4 group. (d-e) Immunohistochemical staining of Ki-67 indicated that tumor proliferation was remarkably reduced in the sh-NXPH4 group. Scale bar: 100 and 200 μm. NC: negative group; sh: short hairpin. *p < 0.05, **p < 0.01,***p < 0.001.

To verify the effect of NXPH4 depletion on BCa tumorigenesis *in vivo*. T24 cells transfected with sh-NXPH4 or sh-NC were injected subcutaneously into nude mice. As presented in Figure 8(a), tumors implanted in sh-NXPH4 group were smaller. Additionally, the tumor volume and tumor weight were lower in sh-NXPH4 group (Figure 8(b,c)). Immunohistochemical staining of Ki-67 indicated that tumor proliferation was remarkably reduced in the sh-NXPH4 group (Figure 8(d,e)).

## Discussion

BCa is one of the most prevalent tumors in the urinary system, characterized by a high invasiveness and recurrence rate [30]. Although conventional treatments such as surgery and chemotherapy can be applied, the prognosis of patients is still unsatisfactory. Therefore, further research into BCa is becoming increasingly urgent, with particular attention being devoted to screening biomarkers that can aid in early detection, risk stratification, and identification of appropriate interventions [31].

The NXPH family consists of five main protein functional structural domains: the N-terminal hydrophobic signal peptide, the N-terminal variable structural domain, a glycosylation-modified central structural domain, the linker region, and a highly conserved linker sequence containing 6 cysteine residues at the C-terminus. Human NXPH proteins are relatively evolutionarily conserved, with four family members, NXPH1, 2, 3 and 4, which have highly similar C-terminal protein sequences but more divergent N-terminal protein sequences [4,32,33]. It has been reported that NXPH1 and 2 are highly expressed in tumor tissues of pancreatic ductal adenocarcinoma with higher malignancy [8]. Specially, NXPH4 is gradually being discovered for its pro-cancer role. In the present study, we found that NXPH4 was highly expressed in BCa tissues by using bioinformatics methods. The expression of NXPH4 in BCa tissues was found to be higher than that in normal tissues, and the expression level of NXPH4 was correlated with clinicopathological characteristics of BCa patients. Our results showed that patients with high NXPH4 expression in BCa had shorter survival time and were more likely to progress and NXPH4 could be used as independent risk factor for survival.

There is growing evidence showed that immune microenvironment play an extremely important role in tumorigenesis. Tumor immunity is closely related to cell growth and any imbalance in the immune microenvironment can accelerate the progression of cancer. Computer algorithms for transcriptomic data are now being widely used to determine dynamic immune components in TME of tumor patients [20,34]. In addition, the study of tumor immune microenvironment and tumor development has received much attention from researchers [35]. Meanwhile, substantial evidences have pointed out characterizing the tumor immune microenvironment can identify new prognostic and predictive biomarkers, which are important for new therapeutic strategies development, and may be used to guide the first-line treatment of tumors [36,37]. In BCa, previous studies have not only demonstrated the key role played by TME components in tumor progression, but also revealed the malignancy of BCa could be determined based on its dynamic changes [38]. We applied the CIBERSORT method to calculate the proportion of immune cells infiltration and the ratio of immune/mesenchymal components in BCa samples from the TCGA database and validated a prognostic biomarker NXPH4 associated with immune regulation of TME in BCa.

Next, we further validated the expression of NXPH4 in BCa tissue specimens and cell lines. In comparison with similar studies published so far, we performed a more adequate validation [39]. The immunohistochemistry results suggested that the expression of NXPH4 was significantly elevated in tumor tissue specimens. The mRNA expression levels detected in the tissue specimens were also generally consistent with the above results. For *in vitro* experiments, we focused on the function of NXPH4 in BCa and demonstrated that cell proliferation and colony formation were significantly inhibited after knocking down NXPH4, implying that NXPH4 has a tumor-promoting effect. *In vivo* study further confirmed that knock-down NXPH4 could inhibited BCa growth. Also, further investigation on the biological pathway of NXPH4 in regulating BCa growth was needed.

## Conclusion

Overall, the present study identified NXPH4 as a novel molecular marker of prognosis associated with immune cell infiltration in BCa. These findings may provide new insights into the prognostic assessment of BCa and inform further studies of tumor immunity in BCa.

## Supplementary Material

Supplemental Material

## Data Availability

The authors declare that the data supporting the findings of the current study are provided in the article.
