# Supplementary material for: Neurexophilin 4 is a prognostic biomarker correlated with immune infiltration in bladder cancer
Source: Bioengineered. 2022 Jun 26;13(5):13986–99. doi: 10.1080/21655979.2022.2085284 (PMC9276049; doi:10.1080/21655979.2022.2085284)
Supplement: Supplemental Material [file KBIE_A_2085284_SM5479.zip › supplementary/Editing Certificate.pdf]

Editing Certificate

This document certifies that the manuscript

NXPH4 Is a Prognostic Biomarker Correlated With Immune Infiltration in Bladder Cancer

prepared by the authors

Xianchao Sun

was edited for proper English language, grammar, punctuation, spelling, and overall style by one or more of the highly qualified native English speaking editors at AJE.

This certificate was issued on **May 20, 2022**

Neither the research content nor the authors' intentions were altered in any way during the editing process. Documents receiving this certification should be English-ready for publication; however, the author has the ability to accept or reject our suggestions and changes. To verify the final AJE edited version, please visit our verification page at aje.com/certificate.
If you have any questions or concerns about this edited document, please contact AJE at support@aje.com.

AJE provides a range of editing, translation, and manuscript services for researchers and publishers around the world.
For more information about our company, services, and partner discounts, please visit aje.com.
