# Supplementary material for: Neurexophilin 4 is a prognostic biomarker correlated with immune infiltration in bladder cancer
Source: Bioengineered. 2022 Jun 26;13(5):13986–99. doi: 10.1080/21655979.2022.2085284 (PMC9276049; doi:10.1080/21655979.2022.2085284)
Supplement: Supplemental Material [file KBIE_A_2085284_SM5479.zip › supplementary/Table S1.docx]

**Table S1. Clinical characteristics of bladder cancer patients from TCGA cohort.**

| Clinical characteristics | TCGA-BLCA |
| --- | --- |
| Age at diagnosis (year)  ≤ 65  ＞ 65  Gender  Male  Female  Grade  Low grade  High grade  T stage  T0-1  T2  T3  T4  N stage  N0  N1  N2  N3  M stage  M0  M1  Stage  Ⅰ  Ⅱ  Ⅲ  Ⅳ | 162  250  304  108  21  388  4  120  196  60  239  47  76  8  196  11  2  131  141  136 |
