# Supplementary material for: Neurexophilin 4 is a prognostic biomarker correlated with immune infiltration in bladder cancer
Source: Bioengineered. 2022 Jun 26;13(5):13986–99. doi: 10.1080/21655979.2022.2085284 (PMC9276049; doi:10.1080/21655979.2022.2085284)
Supplement: Supplemental Material [file KBIE_A_2085284_SM5479.zip › supplementary/Table S2.docx]

**Table S2. Clinical characteristics of 67 bladder cancer patients.**

| Clinical characteristics | Enrolled patients |
| --- | --- |
| Age  ≤ 65  ＞ 65  Gender  Male  Female  Grade  Low grade  High grade  T stage  T1  T2  T3  T4  N stage  N0  N1 | 20  47  55  12  38  29  15  25  23  4  52  15 |
