# Supplementary material for: Neurexophilin 4 is a prognostic biomarker correlated with immune infiltration in bladder cancer
Source: Bioengineered. 2022 Jun 26;13(5):13986–99. doi: 10.1080/21655979.2022.2085284 (PMC9276049; doi:10.1080/21655979.2022.2085284)
Supplement: Supplemental Material [file KBIE_A_2085284_SM5479.zip › supplementary/Table S3.docx]

**Table S3: Primer information.**

Primer sequence.

| **Gene Name** | **Primer Sequence** |
| --- | --- |
| **GAPDH** | F: AATGGGCAGCCGTTAGGAAA  R: GCCCAATACGACCAAATCAGAG |
| **NXPH4** | F: CTCCTTAGGAAGGCCGTCAG  R: CAGCAGCGAAAACTTGAGGG |

F: Forward; R: Reverse.
