# Supplementary figures and images for: Neurexophilin 4 is a prognostic biomarker correlated with immune infiltration in bladder cancer
Source: Bioengineered. 2022 Jun 26;13(5):13986–99. doi: 10.1080/21655979.2022.2085284 (PMC9276049; doi:10.1080/21655979.2022.2085284)

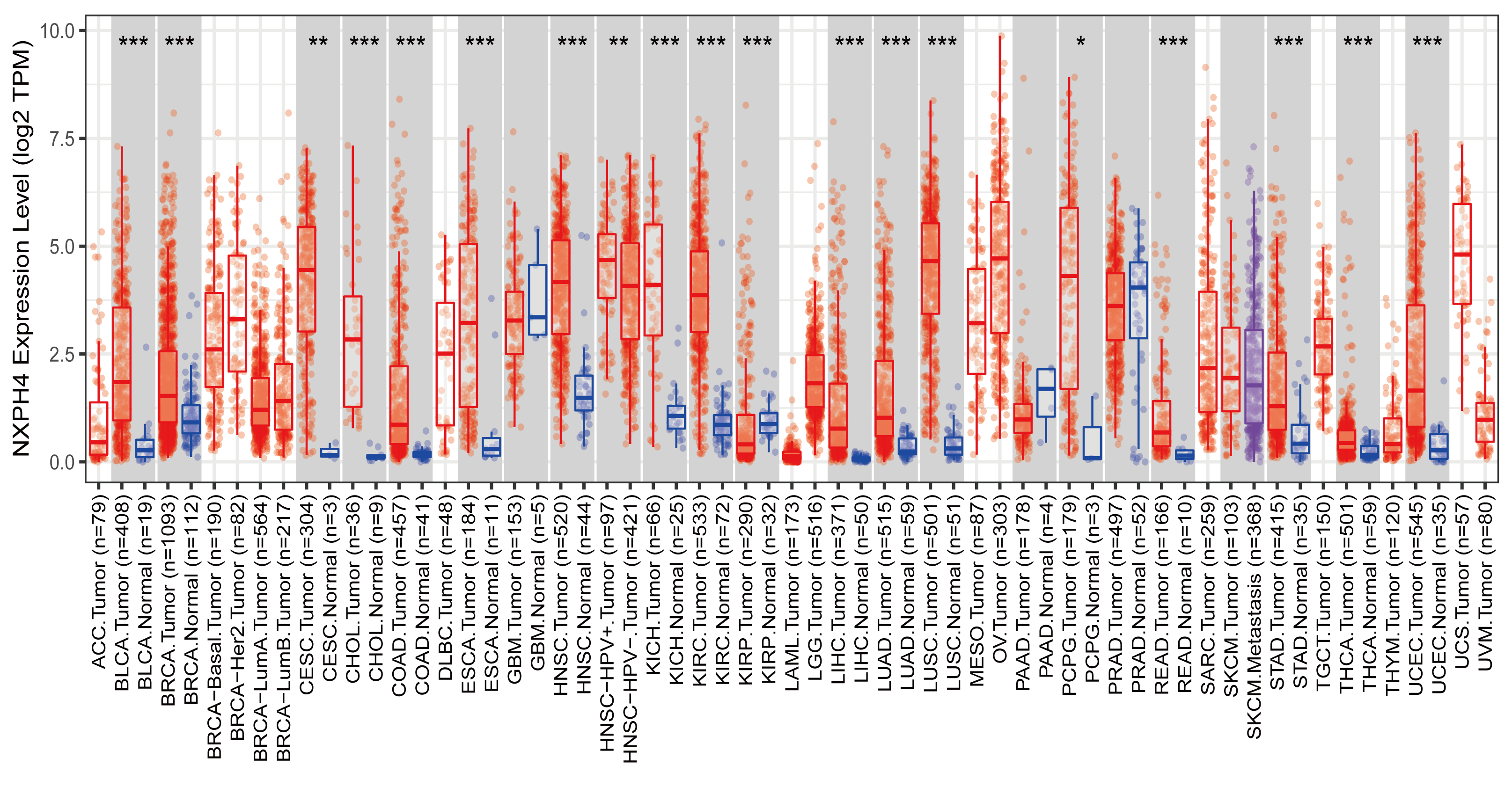

Supplement: Supplemental Material [file KBIE_A_2085284_SM5479.zip › supplementary/Figure S1.tif]

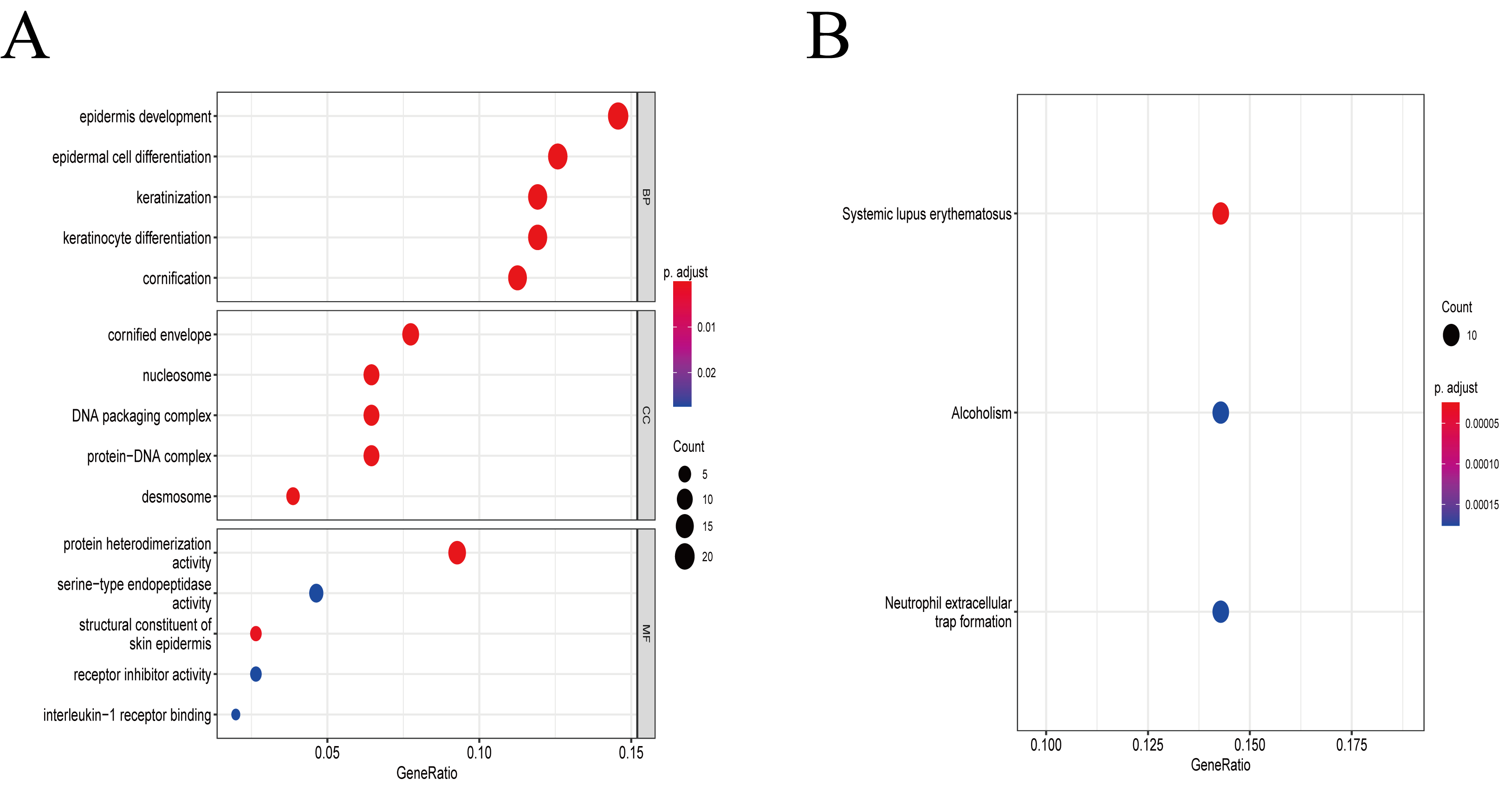

Supplement: Supplemental Material [file KBIE_A_2085284_SM5479.zip › supplementary/Figure S2.tif]

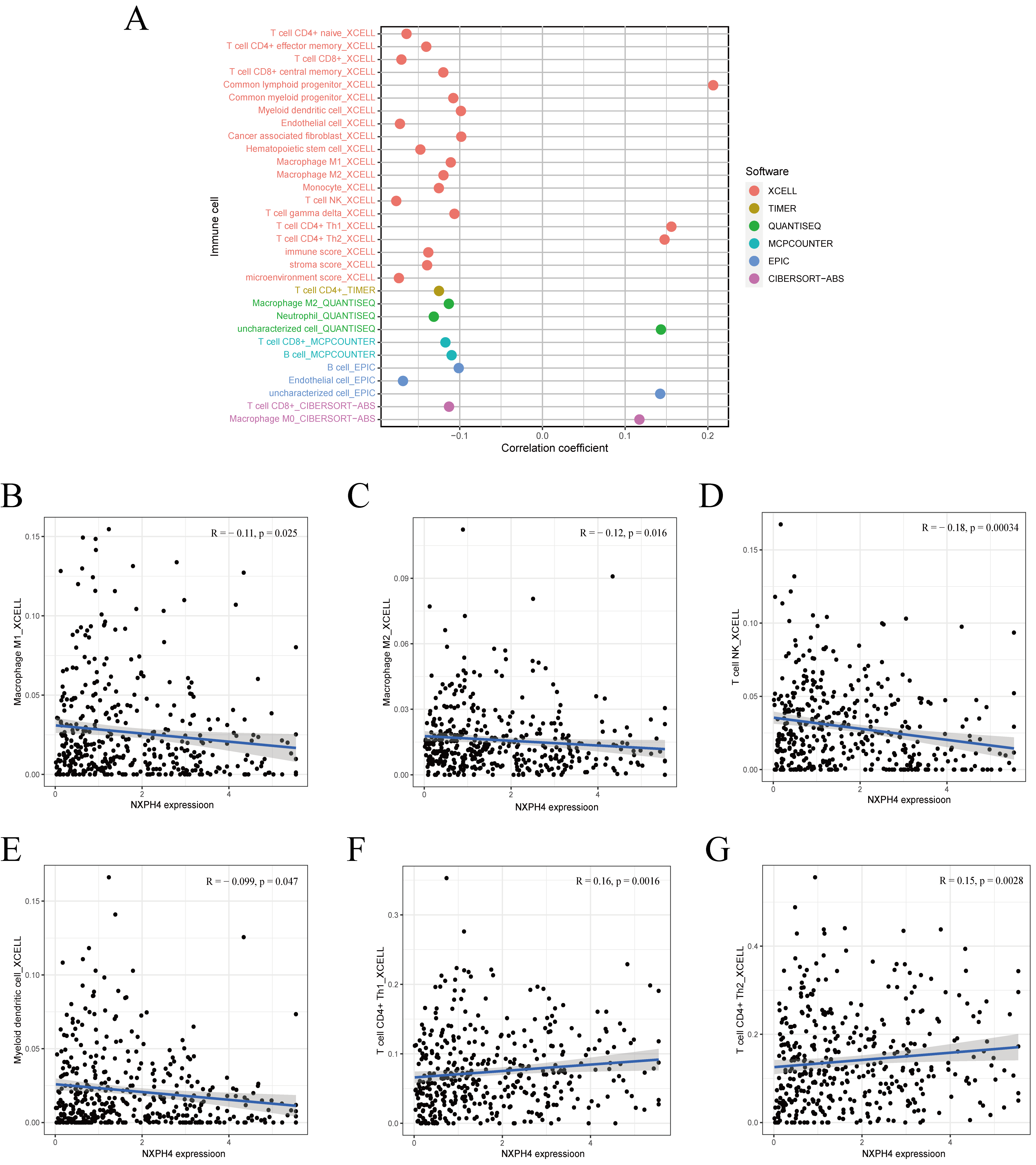

Supplement: Supplemental Material [file KBIE_A_2085284_SM5479.zip › supplementary/Figure S3.tif]

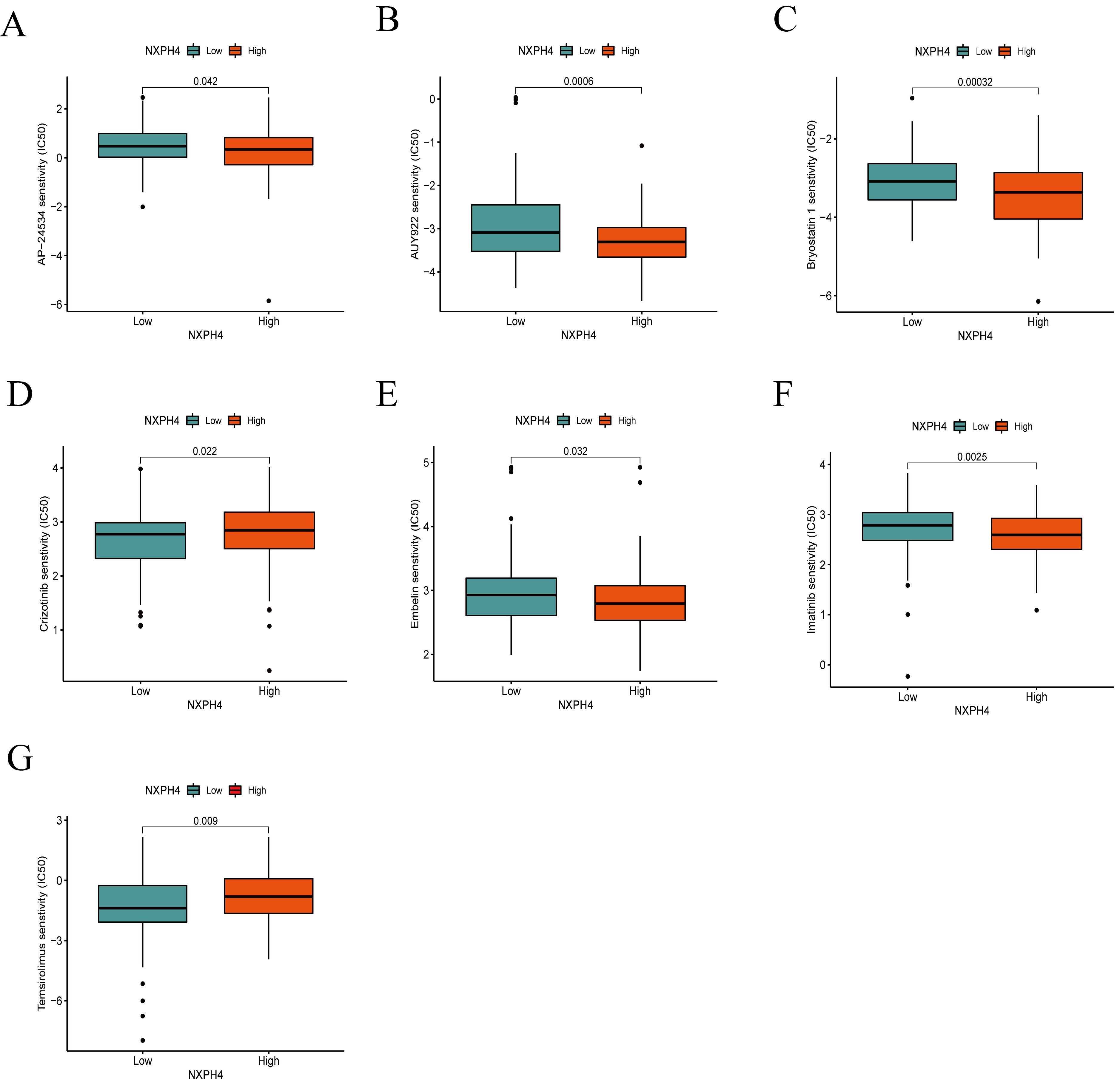

Supplement: Supplemental Material [file KBIE_A_2085284_SM5479.zip › supplementary/Figure S4.tif]
